# Supplementary material for: Simulated Microgravity Inhibits the Proliferation of Chang Liver Cells by Attenuation of the Major Cell Cycle Regulators and Cytoskeletal Proteins
Source: Int J Mol Sci. 2021 Apr 27;22(9):4550. doi: 10.3390/ijms22094550 (PMC8123698; doi:10.3390/ijms22094550)
Supplement: Supplementary file 1 [file ijms-22-04550-s001.zip › ijms-1189606-supplementary.pdf]

Supporting Information

1. Western blot

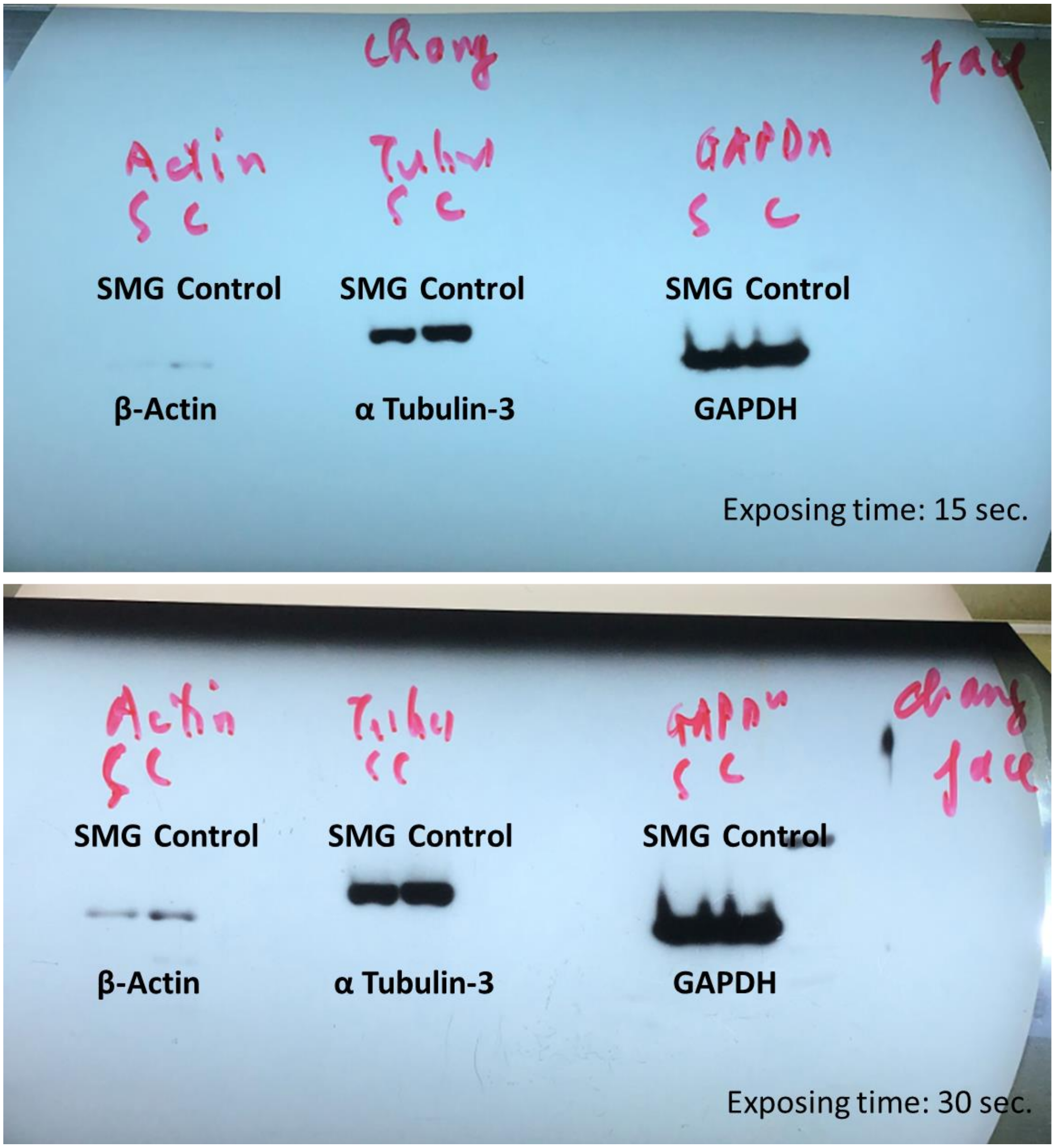

Supplementary Figure S1. X-ray film exposure of β-Actin, α-Tubulin 3 and GAPDH.

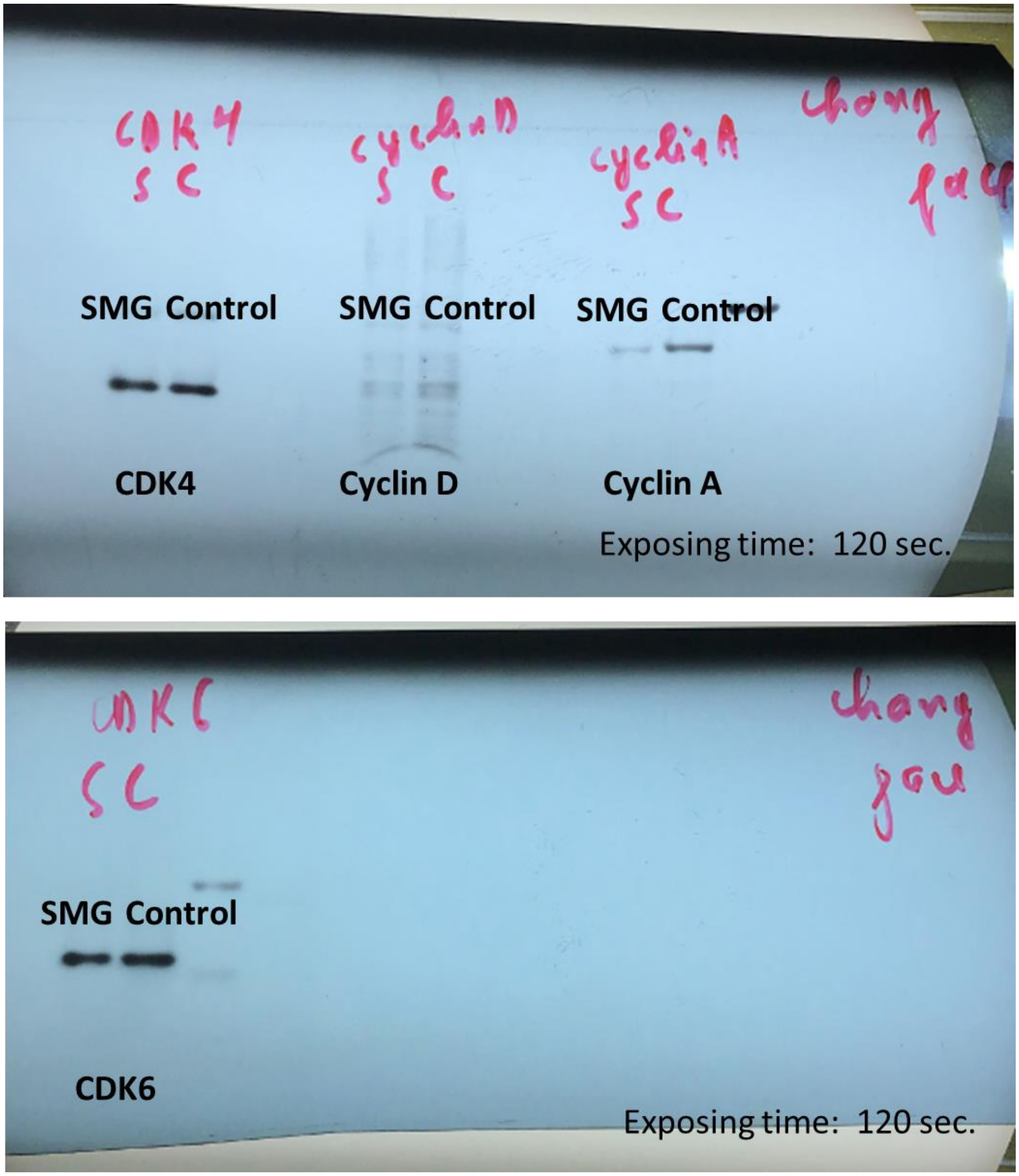

Supplementary Figure S2. X-ray film exposure of CDK4, Cyclin D, Cyclin A, CDK6.

2. 3D clinostat simulation

CCL-13 cells were cultured in T-25 flask. Flasks were filled with culture medium (Supplementary Figure S3A), and the filter-cap was slowly screwed to avoid the formation of bubbles (Supplementary Figure S3B). Flasks were fixed in stage of inner frame of 3D clinostat (Supplementary Figure S3C). 3D clinostat was located in the lower tray in CO_2 incubator, while flasks from the control group were put in the upper tray (Supplementary Figure S3D). Both frames of 3D clinostat rotate constantly with a speed of ~0.105 rad/s and a maximum radius from the center of rotation of 0.05 m. The peak acceleration at the "worst location" of a sample is about 1.3×10^{-3} g. The control group was treated with 1 g. After 72 h, cells were immediately collected for the downstream experiments.

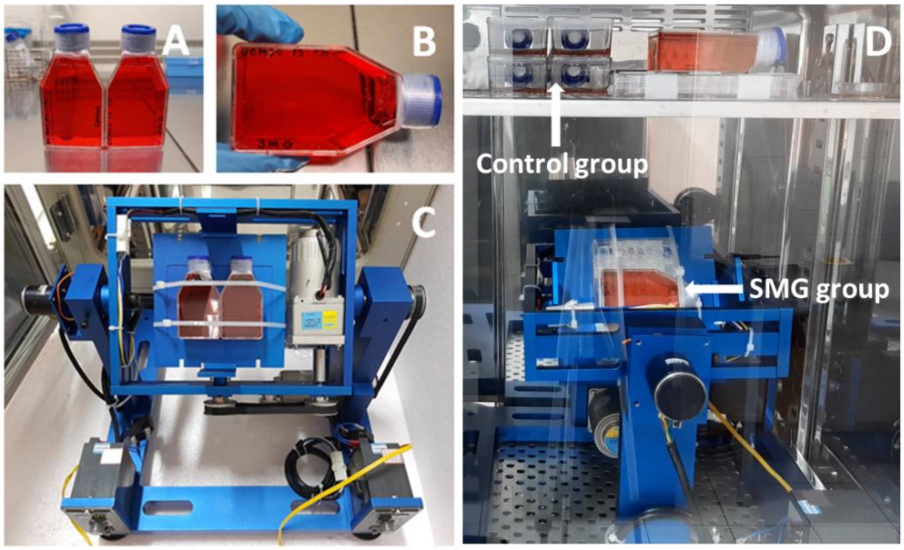

Supplementary Figure S3. The 3D clinostat preparation and operation. (A) T-25 flask was filled up medium and sealed by parafilm tape. (B) Bubbles checking in flask. (C). T-25 flasks were prepared in stage of 3D clinostat. (D) 3D clinostat operation in CO_2 incubator.

Table S1. Cell number counting by Cell Cycle App. of Cytell Microscope.

Well	Cell number/well	
	Control	SMG
1	8664	6615
2	10565	6981
3	9906	6034
4	9947	6333
5	9988	8445
6	10378	3340
7	9598	6634
8	9791	8382
9	10600	7310
10	10308	8310
11	10652	8600
12	10810	8117

Table S2. WST-1 assay of Chang liver cells.

Well	O.D. value measurement	
	Control	SMG
1	7.72E-01	6.37E-01
2	7.48E-01	7.88E-01
3	7.62E-01	7.41E-01
4	7.58E-01	6.05E-01
5	7.78E-01	5.71E-01
6	7.46E-01	6.09E-01
7	7.80E-01	5.83E-01
8	7.41E-01	6.73E-01
9	7.42E-01	5.70E-01
10	7.50E-01	6.50E-01
11	7.41E-01	5.65E-01
12	8.11E-01	4.37E-01

Table S3. Cell cycle analysis (by Flow cytometry).

Group	No.	Cell cycle progression		
		G0/G1 (%)	S (%)	G2/M (%)
Control	1	86.30	11.90	1.00
	2	87.10	11.10	0.90
	3	86.90	11.40	1.00
	4	87.40	10.80	1.00
SMG	1	91.70	6.30	0.60
	2	90.70	7.00	0.70
	3	90.00	7.90	0.60
	4	90.00	7.50	0.80

Table S4. Apoptosis analysis (by Flow cytometry).

Group	No.	Necrosis	Viability	Early apoptosis	Late apoptosis
Control	1	0.4	98.8	0.4	0.4
	2	0.4	98.9	0.4	0.4
	3	0.3	98.9	0.3	0.4
	4	0.4	98.7	0.3	0.3
SMG	1	0.1	99.1	0.5	0.3
	2	0.2	99.0	0.4	0.4
	3	0.2	99.0	0.4	0.3
	4	0.2	99.2	0.3	0.2

Table S5. FSC value (x 10^6).

No.	Control	SMG
1	8.98	9.99
2	8.83	9.91
3	8.78	9.81
4	8.66	9.71
5	8.66	9.58

Table S6. Measurement of nuclear area.

Well	Nuclear area (μm^2)	
	Control	SMG
1	246	231
2	253	234
3	252	234
4	253	239
5	258	242
6	263	254
7	253	254
8	253	248
9	255	257
10	254	251
11	264	255
12	253	235

Table S7. Measurement of nuclear shape value.

Well	Nulear shape value	
	**Control**	**SMG**
1	0.87	0.89
2	0.86	0.88
3	0.89	0.87
4	0.88	0.86
5	0.88	0.88
6	0.86	0.83
7	0.88	0.87
8	0.87	0.87
9	0.88	0.88
10	0.87	0.86
11	0.86	0.88
12	0.88	0.89
